# Extended spectrum-beta-lactamase producing Enterobacteriaceae causing nosocomial infection in a tertiary care hospital, Nepal

**DOI:** 10.1186/2047-2994-4-S1-P122

**Published:** 2015-06-16

**Authors:** MK Sah

**Affiliations:** 1 Department of Clinical Microbiology, Kathmandu University, Kantipur Dental College Teaching Hospital & Research Center, Basundhara, Kathmandu, Nepal

## Introduction

The emergence and spread of resistance in Enterobacteriaceae are complicating the treatment of serious nosocomial infections and threatening to create species resistant to most currently available antimicrobial agents. Extended spectrum β-lactamase (ESBL)-producing Enterobacteriaceae causing nosocomial infection pose unique challenges to clinical microbiologists, clinicians, infection control professionals and antibacterial-discovery scientists in Nepal.

## Objectives

The study was aimed to determine the ESBL-producing Enterobacteriaceae accountable for nosocomial infection.

## Methods

The study was conducted at Tribhuvan University Teaching Hospital (TUTH), a 750 bedded tertiary care referral hospital located at Kathmandu, Nepal. A total of one hundred fifty nine bacterial isolates causing nosocomial infection were studied over a period of one year from March 2011 to February 2012 as described by American Society for Microbiology (ASM). Antibiotic susceptibility testing was performed by the Kirby-Bauer Disk Diffusion technique as recommended by Clinical and Laboratory Standards Institute (CLSI). A combination disk method was done for the detection of ESBL-producing isolates according to the guidelines of CLSI. Data were analyzed using SPSS 17.0 software and interpreted according to frequency distribution and percentage.

## Results

*Escherichia coli* 61.6% (n=98) was found to more predominant which was followed by *Klebsiella pneumoniae* 31.4% (n=50), *Citrobacter freundii* 5.7% (n=9), and *Morganella morgannii* 1.3% (n=2). The prevalence of ESBL was 23.9% (n=38). Among the ESBL producer *Klebsiella pneumoniae* was found to be predominant 26% (n=13) which was followed by *E.coli* 24.5% (n=24) and *C. freundii* 12.5% (n=1).

## Conclusion

It is clear that high prevalence of bacterial strains producing ESBL in our hospital which prompts a special attention for the management of such patients as well as urgent need for implementation of infection control strategies to prevent the dissemination of such strains. ESBL detection should be routinely performed in clinical laboratory as false reporting would results in treatment failure despite in vitro sensitivity.

## Disclosure of interest

None declared.

